# Digital Behavioral Therapy Improves Outcome in Patients With Axial Spondyloarthritis and Persistent Pain: A Randomized Controlled Trial

**DOI:** 10.1002/acr.25679

**Published:** 2025-11-18

**Authors:** David Kiefer, Yade Sonkaya, Dietmar Krause, Markus Voglau, Bernhard Mintrop, Imke Redeker, Xenofon Baraliakos, Uta Kiltz

**Affiliations:** ^1^ Ruhr‐Universität Bochum Bochum Germany; ^2^ Rheumazentrum Ruhrgebiet Herne Germany; ^3^ Private rheumatology practice Gladbeck Germany; ^4^ Private rheumatology practice Oldenburg Germany; ^5^ Private rheumatology practice Hattingen Germany

## Abstract

**Objective:**

Axial spondyloarthritis (axSpA) is often associated with persistent pain despite effective anti‐inflammatory treatment. Digital health applications (DHAs) provide innovative approaches to address multidimensional aspects of persistent pain through psychological and behavioral strategies. The aim of this study was to assess the impact of a DHA using acceptance and commitment therapy (ACT) on disease outcomes, including the West Haven‐Yale Multidimensional Pain Inventory (MPI), in patients with axSpA experiencing persistent pain despite stable pharmacological therapy.

**Methods:**

This unblinded, multicentric, randomized controlled trial compared an intervention group (IG) receiving the ACT app with a standard of care (SOC) group. The ACT app provided behavioral therapy. The primary outcome was MPI pain‐related life interference; secondary outcomes included pain severity, affective distress, and other patient‐reported outcomes after 12 weeks. Linear models estimating the effect of the ACT app on the change of MPI pain‐related life interference and affective distress were calculated.

**Results:**

A total of 136 patients were randomized to IG (n = 73) with the ACT app and SOC (n = 63) without the ACT app. In the IG, 44 actively used the ACT app. All lessons in the ACT app were completed by 19 IG patients (43%). Baseline characteristics, including MPI scores, were comparable between groups. IG showed a reduction in pain‐related life interference as well as in other outcomes. The improvements in pain‐related life interference (β with −0.36, 95% confidence interval [CI]: −0.73 to 0.01) and affective distress related to the disease (−0.4; 95% CI −0.84 to 0.03) were greater compared with SOC.

**Conclusion:**

The ACT app demonstrated a meaningful reduction in pain‐related life interference, supporting that DHAs might become a complementary tool in managing pain for patients with axSpA. Studies about improving adherence to DHAs are warranted.

## INTRODUCTION

Axial spondyloarthritis (axSpA) is a chronic inflammatory disease primarily affecting the axial skeleton, often leading to pain, stiffness, and functional limitations.^1^ Over the past decades, advancements in pharmacologic therapies, including biologic (b) and targeted synthetic (ts) disease‐modifying antirheumatic drugs (DMARDs) targeting inflammatory pathways, have significantly improved the outcome of patients.^2^ However, a notable subset of patients with axSpA continues to report persistent pain despite effective anti‐inflammatory treatment.^3^ As a frequently occurring symptom reported in patients with axSpA, persistent pain is associated with lower health‐related quality of life (HR‐QoL), fatigue, and functional and work productivity impairments.^4^, ^5^ Therefore, this persistent pain presents a considerable challenge in clinical practice. The typical inflammatory back pain is a key symptom of patients with axSpA.^6^ However, the presence of back pain may reflect disease activity but can also potentially be caused by secondary components, such as coping mechanisms, as well as physical and psychological comorbidities.^7^

**SIGNIFICANCE & INNOVATIONS**

- Digital health applications may have the potential to decrease the impact of pain on daily life for patients with axial spondyloarthritis.
- Digital interventions based on acceptance and commitment therapy may improve health‐related quality of life for those with axial spondyloarthritis.
- Digital health applications might serve as a useful addition to standard care in pain management.
- Technical feasibility and usability ratings for the app were generally positive, but long‐term adherence remains a challenge.

Pain management in patients with axSpA primarily involves pharmacotherapy combined with nonpharmacologic treatments, such as exercise, psychological interventions, and self‐management support.^2^ Particularly in patients with predominantly nociplastic and somatoform pain components (eg, fibromyalgia), behavioral therapy approaches play a central role. Various clinical studies have demonstrated that within the framework of multimodal treatment concepts, behavioral therapy measures constitute a key pillar of this approach.^8^ This is also reflected in the current recommendations by EULAR for fibromyalgia, which endorse cognitive behavioral therapy (CBT) as an effective treatment.^9^ Initial approaches to digital solutions have already been investigated and published in clinical studies.^10^ Furthermore, the use of electronic health apps has been shown to effectively reduce pain intensity and its associated interference for patients with chronic back pain.^11^ These applications often integrate evidence‐based psychological therapy and CBT, such as acceptance and commitment therapy (ACT), to address the complex interplay of physical and psychological factors contributing to pain.^12^ By fostering the acceptance of pain, enhancing psychological flexibility, and promoting engagement in meaningful activities, digital health applications (DHAs) hold the potential to improve multidimensional pain outcomes and HR‐QoL.^13^, ^14^

In Germany, DHAs are being reimbursed by statutory health insurance if they have been approved by the German Federal Institute for Drugs and Medical Devices (BfArM).^15^ In March 2025, 68 DHAs in total have been evaluated in terms of efficacy and safety by the regulatory authority. The prescription of the DHA follows a standardized way, which includes an approval step of the health insurance provider, followed by the provision of an activation code to access the DHA.^15^ The costs are fully covered by the patient's health insurance. This system represents the first worldwide opportunity to prescribe DHAs in health care in a manner akin to medical devices or therapeutic aids.

So far, within the field of musculoskeletal disorders, DHAs have only been approved for mental health conditions or orthopedic disorders, reflecting the primary focus of digital therapeutics to date. However, no DHA has been specifically approved for inflammatory and rheumatic and musculoskeletal diseases (RMDs), including axSpA.^15^ Given that pain management and ACT play an important role in the management of axSpA, DHAs have a significant potential for their application in this field. The aim of this study was to assess the impact of a DHA using the ACT on disease outcomes in patients with axSpA experiencing persistent pain despite stable pharmacologic therapy.

## PATIENTS AND METHODS

### Study design

In this multicentric, unblinded, randomized controlled trial, adult patients with a clinical diagnosis of axSpA were eligible for inclusion. This trial was designed with pragmatic elements, aiming to reflect routine clinical practice conditions. Inclusion criteria required a symptom duration of at least 6 months, stable disease control under pharmacologic therapy, and a pain intensity ≥4 on a numerical rating scale (NRS; 0–10, 10 = severe pain) at randomization. Stable disease control was defined as the treating rheumatologist's judgment that no change in immunomodulatory or analgesic therapy was indicated, even if disease activity scores were elevated because of extreme high patient‐reported outcome (PRO) results. This pragmatic approach was chosen to reflect real‐world clinical practice and to target the patient population most likely to benefit from a DHA. Exclusion criteria included language barriers, inability to provide informed consent, planned changes to or conventional or bDMARD therapy, or an inability or unwillingness to use DHAs.

Patients were randomized 1:1 to either an intervention group (IG) or a standard of care (SOC) group and were observed over 12 weeks. Randomization was conducted using a table with random numbers. Only personnel who assigned participants to the intervention had access to the random allocation sequence, which was not the case for personnel who enrolled participants. SOC patients continued to receive treatment as determined by their treating rheumatologist, without the use of the ACT app. IG patients who did not activate the ACT app within 4 weeks received a reminder phone call from the study team to help with obtaining and using the activation code for the DHA. Baseline assessments were repeated if patients activated the DHA 4 weeks after the initial baseline assessment. We defined active app users as those who engaged with the app at least once, and inactive app users as those who either did not use the app at all or were unable to access it because of insurance‐related issues.

### DHA

We used the HelloBetter Ratiopharm Chronischer Schmerz (ACT app), which is an interactive psychological online program designed to sustainably reduce the impact of persistent pain and is approved as a DHA for persistent somatoform pain disorder, persistent pain disorder with somatic and psychological factors, and fibromyalgia.^15^, ^16^ Through evidence‐based psychoeducation provided via texts, videos, and audio content, participants learn effective strategies from ACT.^15^, ^17^ These strategies are reinforced through practical exercises that can be integrated into daily life, helping individuals improve their management of persistent pain and reduce its impact on their QoL.

The HelloBetter app online program is aimed at the long‐term reduction of pain‐related life interference and is available for PCs, tablets, or smartphones. The program consists of seven units, each requiring 45 to 60 minutes to complete. The content was developed by psychologists and psychotherapists with expertise in persistent pain management and digital health, in accordance with established ACT protocols. Its efficacy and safety were evaluated in clinical trials, and it is listed as an approved DHA in the official directory of the BfArM.^15^, ^16^, ^18^, ^19^

### Assessment of axSpA

Data on demographic and disease status as well as information on laboratory results (eg, C‐reactive protein [CRP] and HLA‐B27) and data on current treatment including the nonsteroidal anti‐inflammatory drug (NSAID) usage indices were taken from medical records. Patients responded to a standardized tool of PROs at baseline and after 3 months. Pain assessment included a global pain question (NRS 0–10) and the PainDETECT questionnaire, which ranged from 1 to 24, with a threshold of >18 indicating probable neuropathic pain.^20^ Furthermore, the Multidimensional Pain Inventory (MPI) was assessed.^21^ MPI Part 1, “Pain Experience and Psychosocial Impact,” evaluates the impact of pain across five dimensions using 22 questions:

1. Pain severity: the intensity of the pain experienced.
2. Interference: the extent to which pain disrupts daily life, activities, and overall functioning.
3. Perceived support: the level of emotional and practical support from others.
4. Life control: the degree to which patients feel in control of their pain and life circumstances.
5. Affective distress: the emotional burden associated with living with chronic pain.

Each MPI question is scored on a 7‐point Likert scale (0–6), and subscores are calculated by averaging the responses within each dimension.^21^

Disease activity was measured with the Bath Ankylosing Spondylitis Disease Activity Index (BASDAI) and the Axial Spondyloarthritis Disease Activity Score (ASDAS), which both primarily target nociceptive pain associated with inflammatory activity. Furthermore, disease‐specific assessments included the Bath Ankylosing Spondylitis Functional Index (BASFI), the Physician Global Assessment (PhGA), and the Assessment of Spondyloarthritis International Society Health Index (ASAS‐HI). The Hospital Anxiety and Depression Scale (HADS) was used to evaluate psychological comorbidities. The Fibromyalgia Impact Questionnaire (FIQ) was assessed for pain related to fibromyalgia. The mental component score (MCS) and physical component score (PCS) of the 36‐item Short Form were used to assess physical and mental HR‐QoL. Physical activity levels were quantified using the modified Short Questionnaire to Assess Health‐enhancing physical activity (mSQUASH).

### Assessment of usage of the DHA

Usage of the DHA was provided by self‐report and electronic tools. IG patients provided self‐reports of app usage, detailing the number of completed modules. This was complemented by electronically recorded data to objectively assess engagement, including the percentage of the program completed and the time spent using the app. The mobile app rating scale (MARS) was used to assess app engagement, functionality, aesthetics, and subjective quality, along with a system usability score to quantify the overall user experience. The evaluation resulted in an average value for each section and an average value for the overall app quality between 1 and 5. A value of ≥3 was considered moderate and a value of ≥4 was considered good.^22^ Acceptance of the DHA was assessed using the Net Promoter Score (NRS 0–10): patients with a score from 0 to 6 are considered to be detractors, patients with a score from 7 to 8 are considered to be passives, and patients with a score ≥9 are considered to be promoters.^23^ The Patient Global Impression of Change (PGIC) was used to evaluate patients’ subjective perception of overall change during the study. Patients rate a single‐item questionnaire their perception of change on a 7‐point Likert scale, ranging from “very much improved” (1) to “very much worse” (7).^24^ To identify potential barriers to using the app, we developed a 10‐item study‐specific questionnaire addressing aspects such as usability, technical problems, user adherence, individual needs, data protection, doctor‐patient relationship, digital expertise, implementation into therapy, and costs. Patients rated each item on a scale from 0 (no barrier) to 10 (significant barrier). The System Usability Score is a validated 10‐item questionnaire that evaluates the usability of a system, providing a single score ranging from 0 to 100, with higher scores indicating better usability.^25^

The primary endpoint of the study was change in the West Haven‐Yale MPI dimension of pain‐related life interference after 12 weeks. Secondary endpoints included axSpA PRO measurements and the other dimensions assessed through the MPI.^21^

### Statistical analyses

Differences between baseline and follow‐up were examined using paired *t*‐tests for continuous variables and the McNemar chi‐square test for categorical ones. Baseline adjusted differences in pain‐related interference and emotional distress between active and inactive app users were analyzed using analysis of covariance. A *P* value of <0.05 was considered to be significant.

We assumed for the sample size calculation a sample variance of 1.162 for the MPI after 3 months, a correlation coefficient of 0.5 between pain intensity at baseline and after 3 months, and a mean difference of 0.35 between the intervention and control groups.^13^ Thus, the required number of patients per group is approximately 87, for a two‐sided significance level of α = 0.05 and a power of 1 − β = 0.8.

Given the low rate of missing data (approximately 5% of baseline data), no imputation methods were applied, and complete case analyses were conducted. All analyses were performed in R (version 4.4.2).

The study was conducted across four rheumatology centers in Germany, approved by the Ethical Committee of the Ruhr‐University Bochum, Germany (reference number 23‐7831) and registered in the German Clinical Trials Register (DRKS00037509). Informed written consent was obtained from all patients. All data used in this research are already included in the article.

## RESULTS

### Clinical assessments and app usage

**Figure 1:**
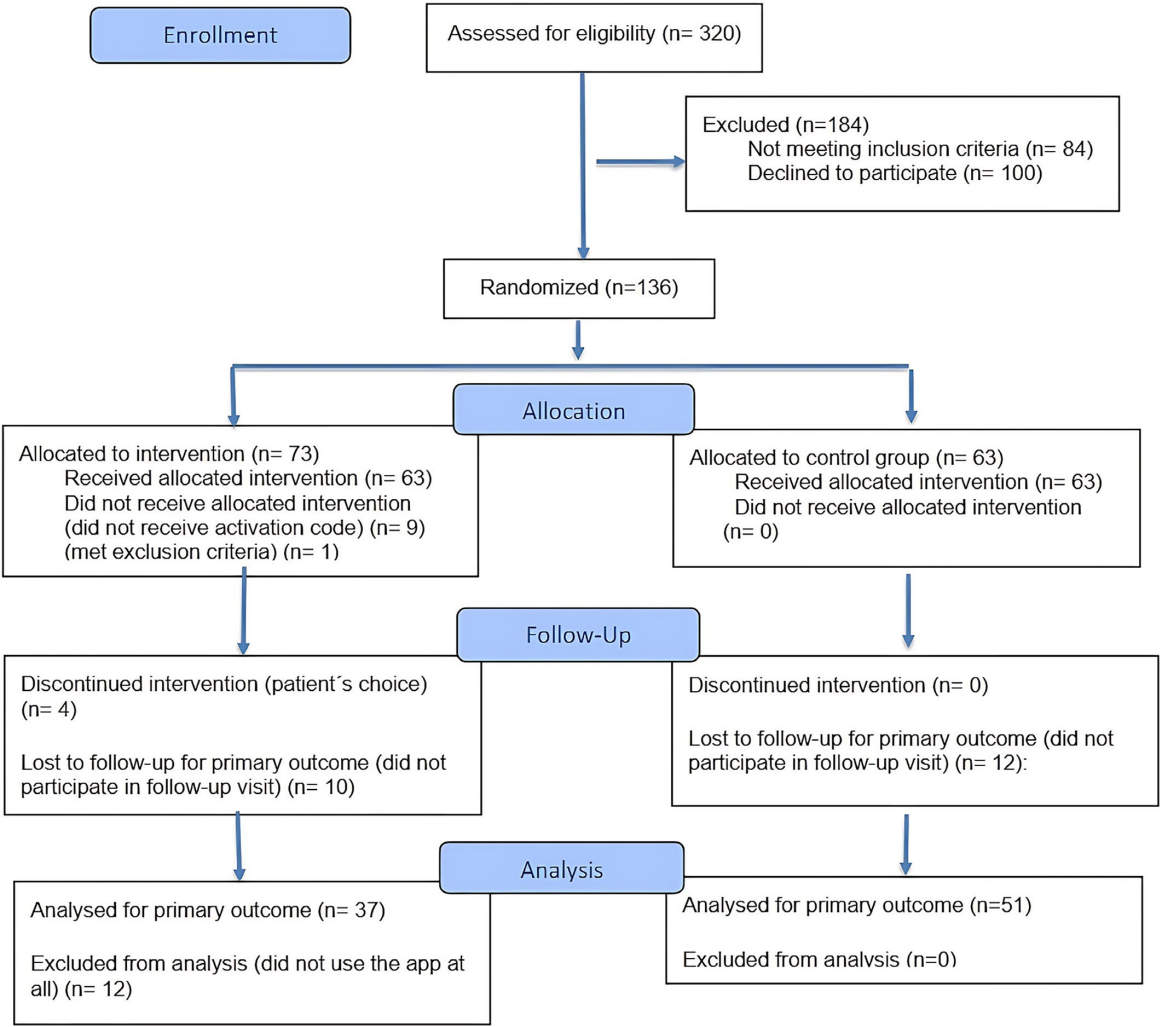
Study flowchart.

A total of 320 patients were screened, and 136 patients were consecutively enrolled in the study, with 73 patients randomized to IG and 63 to SOC between September 2023 and June 2024 (Table 1; Figure 1). The sample size could not be reached within the planned timeframe of 6 months. The extension of the recruitment period had to be discontinued after 4 months for organizational reasons. In line with the elements of a pragmatic design, not all patients randomized to the IG activated or regularly used the ACT app, and levels of engagement varied. Within the IG, 44 patients (60.3%) actively used the ACT app, and 29 (39.7%) were categorized as inactive app users. Of the inactive users, 16 (55.2%) had access to the app but chose not to use it, and 13 (44.8%) did not receive the app activation code from their health insurance provider, leaving them unable to engage with the intervention. At the 12‐week follow‐up, 19 IG patients (43.2%) completed all units in the ACT app, whereas 25 (56.8%) used the ACT app actively but did not complete all seven units. Based on electronic records, active app users completed an average of 70% of the units. (Table 1).

**Table 1:** Demographical characteristics and baseline assessments stratified by group and user activity*

Characteristics	n	All app users (n = 73)	Active users (n = 44)	Inactive users (n = 29)	SOC patients (n = 63)
Age, mean ± SD, y	136	46.8 ± 13	47.3 ± 12	46.2 ± 12.4	50.9 ± 11.4
Gender female, n (%)	136	36 (49.3)	23 (52.3)	13 (44.8)	27 (42.9)
Education, university level, n (%)	123	18 (24.7)	10 (22.7)	8 (27.6)	11 (17.5)
Employment, n (%)	130	51 (68.8)	29 (65.9)	16 (59.3)	35 (55.5)
Time since axSpA symptoms, mean ± SD, y	129	18.2 ± 14.2	17.7 ± 13.8	19.0 ± 15.1	20.3 ± 13.5
Time since axSpA diagnosis, mean ± SD, y	132	13.3 ± 13	13.2 ± 13.7	13.6 ± 12.4	15.2 ± 12.7
BMI, mean ± SD, kg/m^2^	116	28.9 ± 6.7	29.0 ± 6.3	28.6 ± 7.5	28.2 ± 6.7
CRP, mean ± SD, mg/dl	133	0.6 ± 1	0.4 ± 0.8	0.8 ± 1.2	0.6 ± 1.2
HLA‐B 27, n (%)	120	46 (63.0)	25 (67.7)	21 (75.0)	44 (70.0)
Charlson comorbidity index (0–29), mean ± SD	133	1.1 ± 1.2	1.1 ± 1.1	0.9 ± 1.3	1.2 ± 1.3
NSAID index (0–100), mean ± SD	131	18.7 ± 29.2	18.2 ± 31.6	19.6 ± 25.6	19.1 ± 30.4
Patients on csDMARDs, n (%)	136	2 (2.7)	1 (2.3)	1 (3.4)	3 (4.8)
Patients on bDMARDs, n (%)	136	47 (64.4)	29 (65.9)	18 (64.3)	42 (66.6)
Patients on tsDMARDs, n (%)	136	5 (6.8)	4 (9.1)	1 (3.4)	3 (4.8)
Previous bDMARDs, mean ± SD, n	131	1.6 ± 1.4	1.7 ± 1.5	1.5 ± 1.6	1.4 ± 1.1
MPI, mean ± SD					
Pain‐related life interference (0–6)	110	3.4 ± 1.1	3.3 ± 1.3	3.7 ± 0.6	3.6 ± 1.0
Pain severity (0–6)	110	3.4 ± 1.1	3.1 ± 1.2	3.8 ± 0.9	3.84 ± 1.2
Affective distress (0–6)	110	3.2 ± 1.2	3.1 ± 1.3	3.4 ± 1.1	3.2 ± 1.2
Social support (0–6)	110	3.4 ± 1.9	3.3 ± 2.0	3.5 ± 1.7	3.8 ± 1.5
Life control (0–6)	110	3.8 ± 1.2	4.0 ± 1.2	3.2 ± 1.1	3.5 ± 1
ASDAS (0–10), mean ± SD	129	2.7 ± 0.8	2.6 ± 0.8	2.9 ± 0.7	2.9 ± 0.8
BASDAI (0–10), mean ± SD	134	5.1 ± 1.8	5.0 ± 1.9	5.3 ± 1.6	5.4 ± 1.9
PhGA (0–10), mean ± SD	129	3 ± 1.5	2.7 ± 1.6	3.3 ± 1.3	2.9 ± 1.2
BASFI (0–10), mean ± SD	133	4.6 ± 2.4	4.5 ± 2.5	4.8 ± 2.1	4.9 ± 2.3
ASAS Health Index (0–17), mean ± SD	135	8.1 ± 3.5	7.9 ± 3.7	8.3 ± 3.3	8.0 ± 3.8
Pain on NRS (0–10), mean ± SD	134	5.5 ± 1.8	5.4 ± 1.9	5.7 ± 1.7	5.8 ± 1.9
PainDETECT >18, n (%)	135	13 (29.5)	13 (29.5)	6 (21.4)	22 (36.0)
HADS‐D (0–21), mean ± SD	134	7.9 ± 4.3	7.6 ± 4.5	8.3 ± 3.9	9 ± 5.3
HADS‐A (0–21), mean ± SD	134	8.5 ± 4.4	8.3 ± 4.6	9.0 ± 4.1	9 ± 4.3
HADS‐D ≥8, n (%)	134	39 (53.4)	21 (47.7)	18 (62.1)	34 (55.7)
HADS‐A ≥8, n (%)	134	43 (58.9)	24 (54.5)	19 (65.5)	30 (49.2)
FIQ (0–100), mean ± SD	127	49.4 ± 16.1	49.6 ± 15.6	49.2 ± 17.1	52.7 ± 21.3
SF‐36 (PCS) (0–100), mean ± SD	132	33.5 ± 8.3	33.5 ± 9.6	33.5 ± 6.1	32.6 ± 7.4
SF‐36 (MCS) (0–100), mean ± SD	132	41.7 ± 12.2	42.6 ± 12.2	40.4 ± 12.2	42.1 ± 13.1
mSQUASH total activity score, mean ± SD	132	9,483 ± 4,878	9,473 ± 5,156	9,500 ± 4,484	9,137 ± 5,675

* ASAS, Assessment of Spondyloarthritis International Society; ASDAS, Ankylosing Spondylitis Disease Activity Score; axSpA, axial spondyloarthritis; BASDAI, Bath Ankylosing Spondylitis Disease Activity Index; BASFI, Bath Ankylosing Spondylitis Functional Index; bDMARD, biologic disease‐modifying antirheumatic drug; BMI, body mass index; CRP, C‐reactive protein; csDMARD, conventional synthetic DMARD; FIQ, Fibromyalgia Impact Questionnaire; HADS‐A, Hospital Anxiety and Depression Scale – anxiety subscale; HADS‐D, HADS – depression subscale; MCS, mental component summary; MPI, Multidimensional Pain Inventory; mSQUASH, modified Short Questionnaire to Assess Health‐enhancing physical activity; NRS, numerical rating scale; NSAID, nonsteroidal anti‐inflammatory drug; PCS, physical component summary; PhGA, Physician Global Assessment; SF‐36, 36‐item Short Form; SOC, standard of care; tsDMARD, targeted‐synthetic DMARD.

Baseline characteristics, including demographic and clinical data, were comparable between the IG and SOC group. The mean ± SD age was 46.8 ± 13.0 years in the IG and 50.9 ± 11.4 years in the SOC group. Gender distribution was similar, with 49.3% of the IG and 42.9% of the SOC group being female. Time since first symptoms and time since diagnoses were comparable. Importantly, there was no difference in the usage of NSAIDs and b/tsDMARDs or the comorbidity index (Table 1). The CRP level was also comparable.

At baseline, IG and SOC showed similar baseline values of the MPI dimensions. In detail, the mean ± SD results for pain‐related life interference (3.4 ± 1.1 vs 3.6 ± 1.0), pain severity (3.4 ± 1.1 vs 3.8 ± 1.2), affective distress (3.2 ± 1.2 in both groups), social support (3.4 ± 1.9 vs 3.8 ± 1.5), and life control (3.8 ± 1.2 vs 3.5 ± 1.0) were comparable between the IG and SOC group (Table 1). Furthermore, baseline measures for disease activity (ASDAS, BASDAI, and PhGA), physical and global functioning (BASFI, PCS, MCS, and ASAS‐HI), spinal mobility (Bath Ankylosing Spondylitis Metrology Index) and measurements of pain (pain on NRS and Pain DETECT), and physical activity (mSQUASH) as well as Patient Acceptable Symptom State, HADS, FIQ, and Patient‐Activation Measure‐13 results showed no relevant differences between the IG and SOC group (Table 1).

**Figure 2:**
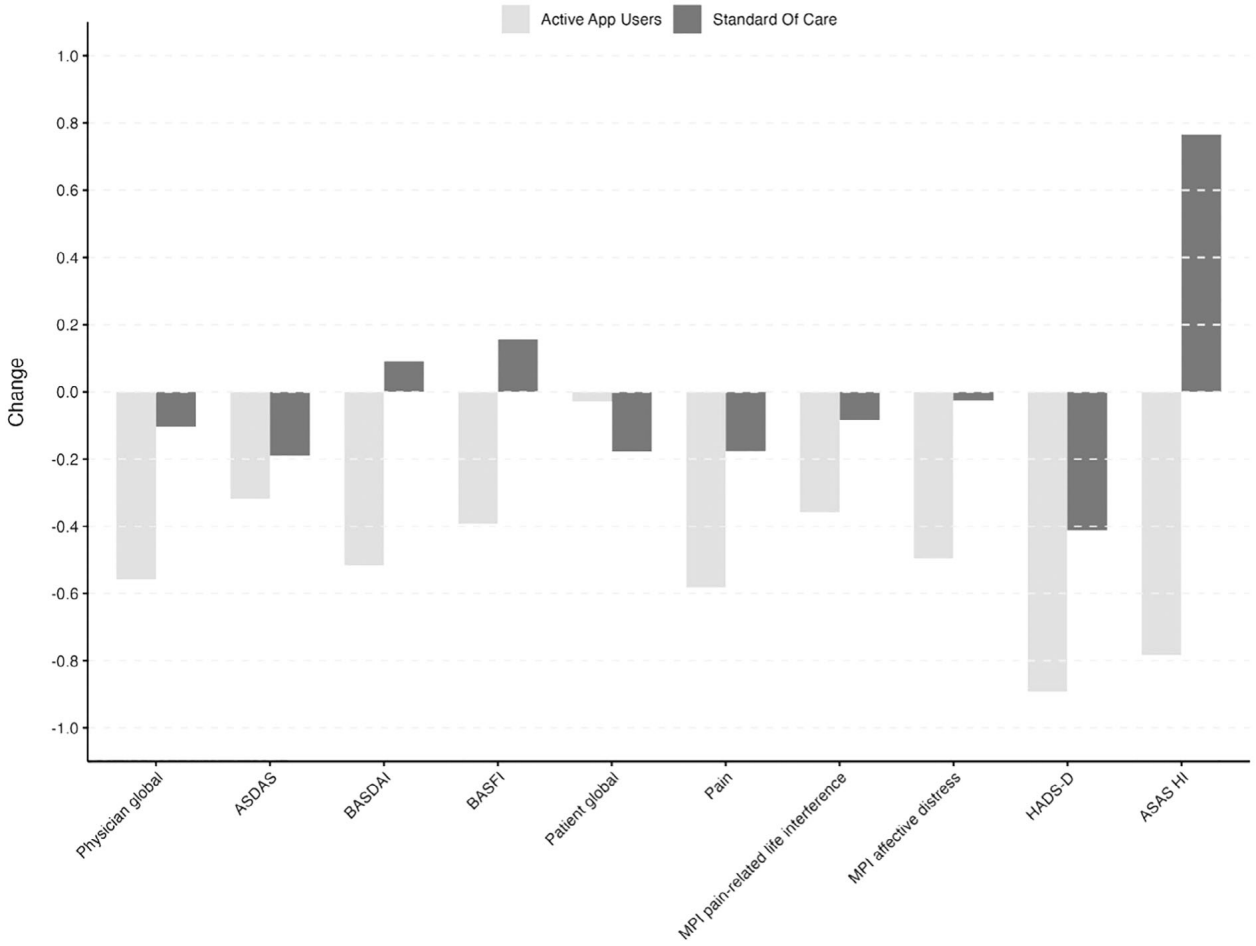
Change of clinical variables stratified by group. Mean change of assessments compared between active app users and standard of care patients. ASAS‐HI, Assessment of Spondyloarthritis International Society Health Index; ASDAS, Ankylosing Spondylitis Disease Activity Score; BASDAI, Bath Ankylosing Spondylitis Disease Activity Index; BASFI, Bath Ankylosing Spondylitis Functional Index; HADS‐D, Hospital Anxiety and Depression Scale – depression subscale; MPI, Multidimensional Pain Inventory.

Improvements in pain‐related life interference (−0.36; 95% confidence interval [CI] −0.73 to 0.01) and affective distress related to the disease (−0.4; 95% CI −0.84 to 0.03) were greater in IG compared with SOC (Table 2). Improvements in most secondary disease‐specific measures were more pronounced in active app users compared with SOC (Table 2; Figure 2). In particular, BASDAI (−0.76; 95% CI −1.3 to −0.25), PhGA (−0.56; 95% CI −0.98 to −0.14), BASFI (−0.65; 95% CI −1.1 to −0.19), PainDETECT (−2.13; 95% CI −4.1 to −0.12), and ASAS‐HI (−1.32; 95% CI −2.3 to −0.35) showed a greater improvement in IG compared with SOC. The percentage of patients with clinically relevant HADS – depression subscale scores (≥8) reduced from 46.5% to 32.4% in IG and from 55.7% to 46.8% in SOC, whereas the percentage of patients with clinically relevant anxiety was only reduced in IG (53.5% to 43.2%) and slightly increased in SOC (49.2% to 53.2%).

**Table 2:** Effect of the digital health application on primary and secondary outcomes (analysis of covariance analyses adjusted for outcome at baseline)*

Outcome	β	95% CI
Pain‐related interference, 0–6	−0.36	−0.73 to 0.01
Emotional Distress, 0–6	−0.40	−0.84 to 0.03
ASDAS, 0–10	−0.09	−0.37 to 0.20
BASDAI, 0–10	−0.76	−1.3 to −0.25
PhGA, 0–10	−0.56	−0.98 to −0.14
BASFI, 0–10	−0.65	−1.1 to −0.19
Pain, NRS 0–10	−0.61	−1.3 to 0.10
PainDETECT, 0–38	−2.13	−4.1 to −0.12
Pain severity (MPI), 0–6	−0.12	−0.48 to 0.25
Social support (MPI), 0–6	0.07	−0.43 to 0.57
Life control (MPI), 0–6	0.05	−0.45 to 0.54
HADS‐D, 0–21	−0.92	−1.9 to 0.07
HADS‐A, 0–21	−0.77	−1.9 to 0.33
FIQ, 0–100	−4.66	−10 to 0.65
ASAS Health Index, 0–17	−1.32	−2.3 to −0.35
SF‐36 (PCS), 0–100	2.15	−0.32 to 4.6
SF‐36 (MCS), 0–100	1.74	−1.5 to 5.0
mSQUASH, total activity score	−475.47	−2,016 to 1,065

* ASAS, Assessment of Spondyloarthritis International Society; ASDAS, Ankylosing Spondylitis Disease Activity Score; BASDAI, Bath Ankylosing Spondylitis Disease Activity Index; BASFI, Bath Ankylosing Spondylitis Functional Index; CI, confidence interval; FIQ, Fibromyalgia Impact Questionnaire; HADS‐A, Hospital Anxiety and Depression Scale – anxiety subscale; HADS‐D, HADS – depression subscale; MCS, mental component summary; MPI, Multidimensional Pain Inventory; mSQUASH, modified Short Questionnaire to Assess Health‐enhancing physical activity; NRS, numerical rating scale; PCS, physical component summary; PhGA, Physician Global Assessment; SF‐36, 36‐item Short Form.

**Figure 3:**
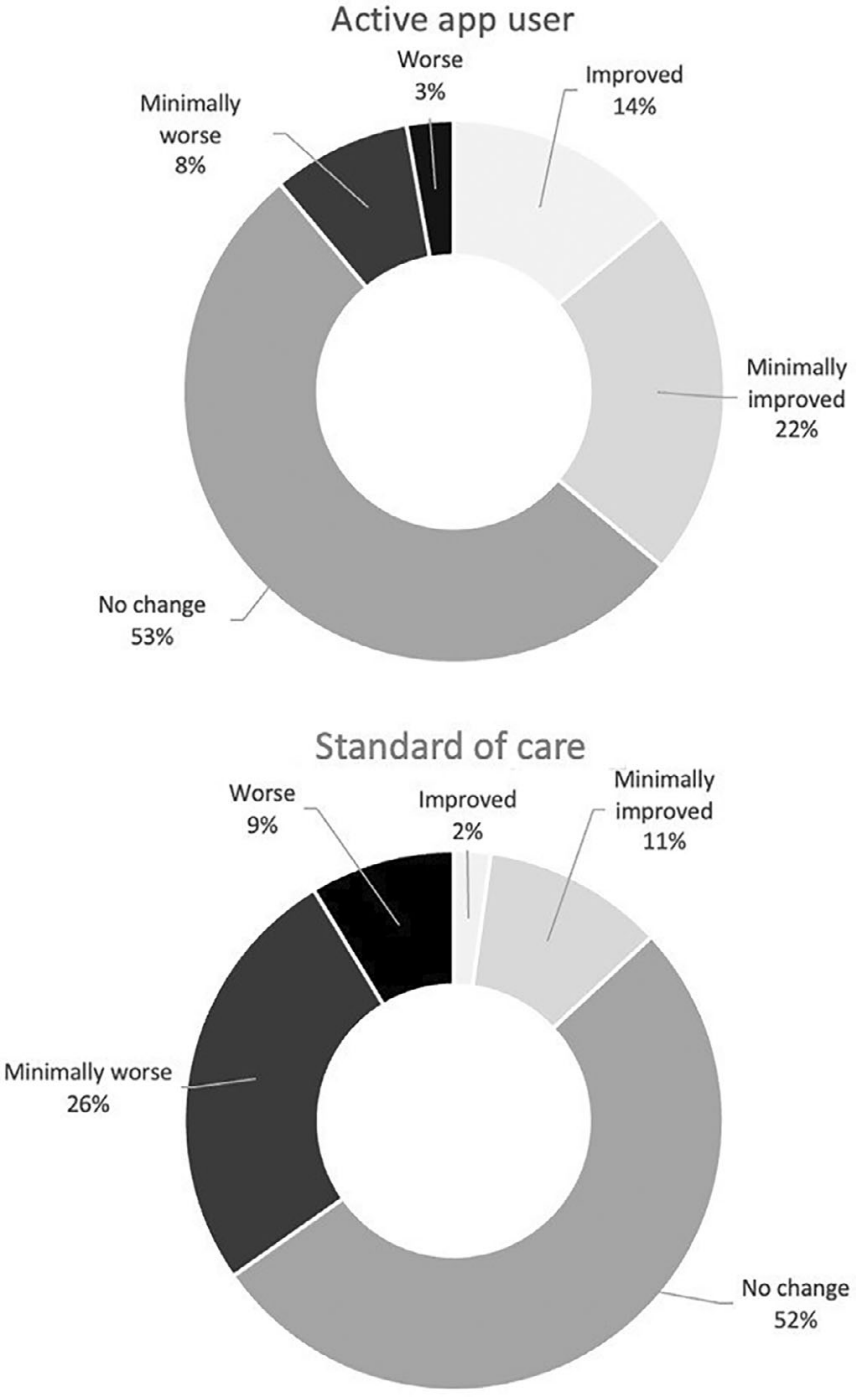
Patient Global Impression of Change categories: comparison between active app users and the standard of care group. All categories are given as percentages. The two categories at the end of the 7‐point Likert scale (“very much improved”/“much improved” and “very much worse”/“much worse”) have been grouped together resulting in five categories ranging from “improved” to “worse.”

A higher proportion of active app users had a subjective perception of improvement (PGIC) over 12 weeks in contrast to SOC patients (36 vs 13%). Conversely, more participants in the SOC group had a subjective perception of worsening compared with active app users (35 vs 11%) (Figure 3).

### App evaluation metrics

**Figure 4:**
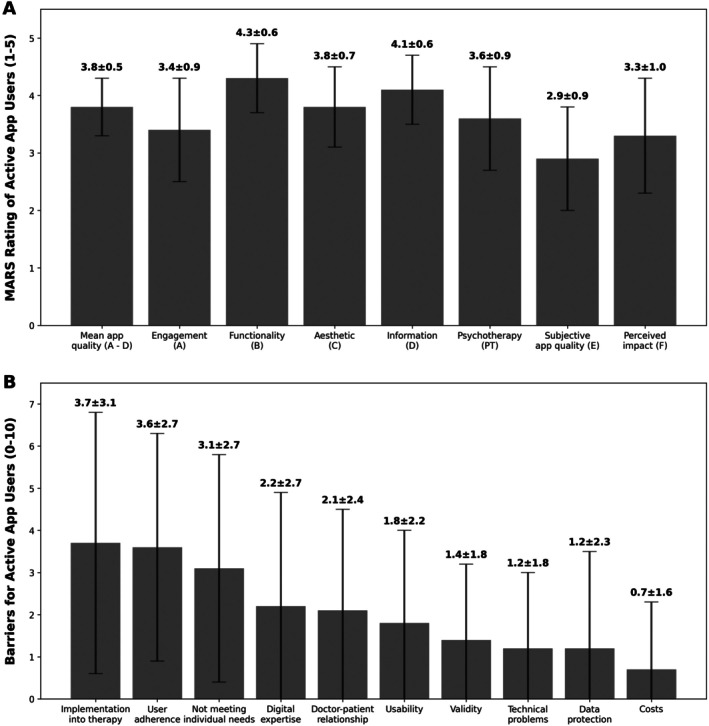
Active app user's (A) MARS rating and (B) barriers to using the app. (A) Mean ± SD values of the overall MARS rating of active app users. (B) Mean ± SD values of the barriers questionnaire (score 1–10) of active app users ranged from the largest to smallest barrier. MARS, mobile app rating scale.

The DHA was promoted by five patients (14.7%), whereas the others were either passive users (n = 9, 26.5%) or detractors (n = 20, 58.9%). The quality of the ACT app was evaluated as being good with a mean ± SD MARS score of 3.8 ± 0.5 (Figure 4A). The best mean ± SD MARS rating was achieved in the functionality domain (4.3 ± 0.7) followed by the information domain (4.1 ± 0.6). In the subjective quality domain, the app achieved the weakest rating, with a mean ± SD score of 2.9 ± 0.9. The effect of the app on patients’ health behavior was rated as moderate with a mean ± SD score of 3.3 ± 1.0. The usability was rated favorable with a mean ± SD score of 73.3 ± 17.0.

We identified minor barriers to the use of the app (Figure 4B). Moderate challenges were related to *implementation into therapy* (3.7 ± 3.1), *user adherence* (3.6 ± 2.7), and the *app not meeting individual needs* (3.1 ± 2.7). Minor barriers included *required digital expertise* (2.2 ± 2.7), *impact on the doctor‐patient relationship* (2.1 ± 2.4), usability (1.8 ± 2.2), *validity* in meeting user expectations (1.4 ± 1.8), *technical problems to access* (1.2 ± 0.8), *data protection concerns* (1.2 ± 2.3) and *costs* (0.7 ± 1.6) (Figure 4B).

## DISCUSSION

Our study investigated the effectiveness of the HelloBetter app, a DHA, in addressing multidimensional pain in patients with axSpA and persistent pain. The primary outcome of the study, the reduction in pain‐related life interference measured by the MPI, indicated a greater improvement in IG compared with SOC. Although the absolute change in pain‐related life interference was small, it may still be clinically relevant in a population with stable disease control under optimized pharmacologic treatment, as even modest improvements can have a meaningful impact when achieved through a low‐risk and easily accessible intervention such as a DHA.

A recent meta‐analysis found that ACT reduced pain intensity after treatment compared with applied relaxation, with a standardized mean difference of −0.26 (95% CI −0.58 to 0.07), consistent with the small change observed in our study.^12^ This is consistent with earlier studies highlighting the efficacy of DHAs in addressing chronic back pain through psychological and behavioral strategies,^26^ whereas there was no effect on neuropathic pain in our study. The observed reduction in emotional distress and improvements in disease‐specific QoL measures and the slight improvement in the PGIC further supports the utility of DHAs in managing complex pain syndromes in axSpA, including nociplastic pain.^27^ ACT has the potential to enhance psychological flexibility and improve patient outcomes by focusing on helping individuals accept their pain and commit to actions that align with their values, thereby reducing the psychological burden associated with chronic conditions.^28^

Studies indicate that ACT can lead to improve several outcomes including physical functioning in patients with axSpA. A recent study demonstrated that ACT‐based digital interventions effectively improve physical functioning.^27^ Moreover, integrating ACT into DHAs offers a new approach to delivering psychological interventions. Digital platforms can provide accessible, cost‐effective, and user‐friendly means for patients to engage with ACT principles, potentially enhancing adherence and engagement.^29^ This is particularly relevant in rheumatology, in which patients may face barriers to accessing traditional in‐person therapy sessions.^30^ The incorporation of ACT and digital health into comprehensive treatment plans for axSpA aligns with current recommendations emphasizing the importance of addressing both the physical and psychological aspects.^31^, ^32^ ACT empowers patients to manage their symptoms more effectively, leading to improved overall well‐being.^13^, ^17^ The minimal improvement observed in ASDAS and BASDAI was marginal and did not reach the threshold for minimal clinical importance. Therefore, we cannot conclude that ACT has an effect on disease activity.^33^, ^34^

Our findings also align with evidence from studies on other digital health solutions, which demonstrated significant reductions in back pain and improved functional outcomes among patients with chronic conditions.^26^, ^27^ Another recent study demonstrated the effectiveness of a digital ACT program in reducing symptom burden and improving PROs in fibromyalgia, a condition often overlapping with axSpA.^35^, ^36^, ^37^, ^38^ Previous evidence supported the use of DHAs in persistent pain management through psychological and behavioral strategies. A recent study highlighted the increasing acceptance and use of digital health technologies among rheumatology patients, emphasizing their utility in enhancing patient‐centered care and improving access to therapeutic interventions.^39^

The SOC group, which followed traditional rheumatologist‐guided care without the use of the app, demonstrated no significant improvements in MPI dimensions. This highlights the limitations of standard care in addressing the multidimensional aspects of persistent pain. By integrating behavioral therapy into its framework, the HelloBetter app provides a more comprehensive approach to pain management, addressing also the psychological components of persistent pain. Other DHAs have similarly shown their utility in bridging the gaps left by conventional care.

The results of this study highlight the transformative potential of DHAs in the clinical management of axSpA. The HelloBetter app's ACT‐based framework aligns with current recommendations for managing RMDs and also recommendations for the management of fibromyalgia and related disorders, which emphasize the importance of psychological and behavioral interventions.^9^, ^40^ By enabling patients to engage with evidence‐based therapies from the comfort of their homes, DHAs remove many of the logistical barriers associated with traditional care models, such as transportation difficulties and time constraints, which are known barriers for patients.^30^ For clinicians, the integration of DHAs offers an opportunity to enhance patient self‐management. Given the limited time available in routine clinical practice to deliver comprehensive ACT, DHAs can help save valuable time during consultations while still enabling effective implementation of ACT principles and improving pain management outcome.^39^, ^41^, ^42^

In addition, the 2021 EULAR recommendations for the implementation of self‐management strategies in patients with inflammatory arthritis and the 2024 EULAR points to consider for patient education in physical activity and self‐management of pain during transitional care state that digital health care can help patients to self‐manage and should be considered for inclusion in supported self‐management where appropriate and available. They also state that patient education during transitional care should consist of a variety of learning formats, including digital health, and that digital solutions seem to be both feasible and beneficial for patients with RMDs, especially for younger patients.^31^, ^32^, ^43^, ^44^

We have to acknowledge that the DHA did not provide a clear benefit for all patients, as perceived improvements were minimal with a moderate perceived positive influence of the DHA on user's life, although technical functionality information quality and usability were rated positively. Adherence to digital interventions remains a critical challenge, with only 43% of participants in the IG completing all lessons in the app. This adherence rate mirrors findings from similar studies involving digital therapeutics, highlighting the need for enhanced user engagement strategies.^45^ Barriers to adherence often include low digital literacy, lack of motivation, and insufficient technical support. In contrast, patients state that they are willing to use digital applications, such as DHAs.^42^, ^46^, ^47^, ^48^ Furthermore, onboarding processes should be optimized, including detailed user education and ongoing technical support, to ensure patients feel confident using the app.^42^, ^48^

In line with this, several challenges were encountered during our study, including the relatively low activation rate of the app within the first 4 weeks, which necessitated follow‐up interventions to assist with onboarding. Moreover, excluding patients who could not access the DHA because of insurer delays may underestimate real‐world barriers.

Furthermore, even when the cost of the HelloBetter app was covered by insurance companies, some patients did not receive an activation code from their insurers. This could represent a potential barrier to the successful implementation and widespread adoption of DHAs in the future. Another limitation of our study is that we were unable to reach the sample size of 87 patients per group. This is based on the high screening failure rate of 42% with a substantial number of patients unwilling to use an app. Another limitation is the unblinded design of the study, which may have influenced both PROs and the interpretation of results. Future studies should aim to incorporate physician‐blinded study designs, as patient blinding is not feasible with DHAs. In addition, the open‐label design may also have introduced expectancy bias in PROs.

The generalizability of our findings to settings outside Germany is limited, as prescription‐based, health insurance–funded DHAs currently exist only in Germany. Nevertheless, other types of digital tools to support patients are available internationally. Unlike many freely available or commercial apps, DHAs are subject to governmental approval by the BfArM and must demonstrate efficacy and safety according to defined regulatory requirements. The underlying therapeutic concept and delivery format, however, may be transferable to other health care systems, particularly where there is increasing interest in integrating evidence‐based digital interventions into routine care. Future research should focus on optimizing the design and functionality of DHAs to enhance user engagement and long‐term adherence and may include gamification.^49^ Economic evaluations are also needed to assess the cost‐effectiveness of DHAs in pain management. Additionally, studies should investigate the long‐term impact of DHAs on disease progression, comorbidities, and overall QoL.

The digital intervention demonstrated reductions in pain‐related life interference and emotional distress in patients with axSpA, supporting its potential as a supplementary tool in pain management. These findings contribute to the growing body of evidence supporting the use of DHAs in rheumatology and underscore their role in addressing the multidimensional nature of persistent pain. By fostering greater accessibility, personalized care, and patient empowerment, DHAs represent a transformative advancement in the management of complex chronic conditions.

## AUTHOR CONTRIBUTIONS

All authors contributed to at least one of the following manuscript preparation roles: conceptualization AND/OR methodology, software, investigation, formal analysis, data curation, visualization, and validation AND drafting or reviewing/editing the final draft. As corresponding author, Dr Kiltz confirms that all authors have provided the final approval of the version to be published and takes responsibility for the affirmations regarding article submission (eg, not under consideration by another journal), the integrity of the data presented, and the statements regarding compliance with institutional review board/Declaration of Helsinki requirements.

## Supporting information


**Disclosure Form**:
